# Diagnosis and management of gout: are the British Society for Rheumatology and National Institute for Health and Care Excellence guidelines both needed?

**DOI:** 10.1093/rap/rkae007

**Published:** 2024-01-22

**Authors:** Edward Roddy, Kelsey M Jordan, Ian Giles

**Affiliations:** School of Medicine, Keele University, Keele, UK; Haywood Academic Rheumatology Centre, Midlands Partnership University NHS Foundation Trust, Stoke-on-Trent, UK; Rheumatology Department, University Hospitals Sussex NHS Foundation Trust, Brighton, UK; Centre for Rheumatology, Division of Medicine, University College London, London, UK

Numerous international guidelines have been developed relating to the diagnosis and/or management of gout, including those published by the ACR [1], EULAR [2, 3] and the American College of Physicians [4]. In the UK, the first British Society for Rheumatology (BSR) gout guideline was published in 2007 and updated in 2017 [5, 6], whereas the National Institute for Health and Care Excellence (NICE) published its guideline for the diagnosis and management of gout in 2022 [7]. Not surprisingly, given that 5 years elapsed between the publication of these two UK guidelines, there are some differences between their recommendations. In this editorial, we compare and contrast the 2017 BSR and 2022 NICE gout guidelines [6, 7] and consider the extent to which the BSR guideline is superseded by the NICE guideline.

Both guidelines followed robust transparent guideline development methods, including a systematic search and review of the literature, and the BSR guideline protocol is accredited by NICE. Multidisciplinary expertise from rheumatology, general practice, nursing, nephrology and people with lived experience of gout contributed to both guidelines. The BSR guideline working group also had representation from clinical pharmacology, whereas the NICE guideline committee included a dietician and a pharmacist. The NICE guideline formally assessed the cost-effectiveness of its recommendations, whereas the BSR guideline included a section on applicability, utility and implementation, including cost implications.

Both guidelines highlighted the importance of providing people with gout with high-quality tailored education and information about gout, its causes and consequences, how to manage flares, and the rationale, aims and use of long-term urate-lowering therapy (ULT), in addition to assessing lifestyle and co-morbidities, including cardiovascular risk factors and chronic kidney disease. With regard to pharmacological therapy, they agreed that people with gout should be made aware of the option of treat-to-target ULT when the diagnosis is first confirmed, and that it should be more strongly recommended to those with frequent flares, tophi, chronic gouty arthritis or chronic kidney disease and those taking diuretics. They also agreed that anti-inflammatory prophylaxis should be offered when initiating ULT and that colchicine is the first-line drug for prophylaxis.

The BSR and NICE guideline recommendations differed in several areas (Table 1). The BSR guideline focused on gout management, whereas the NICE guideline covered both diagnosis and management. For flare management, the BSR guideline recommended NSAIDs or colchicine as first-line options, with oral CS reserved for people who do not tolerate these. In the NICE guideline, NSAIDs, colchicine or oral corticosteroids (CS) was recommended as the first-line treatment for flares, depending on the person’s co-morbidities, co-prescriptions and preferences, reflecting several randomized trials reporting similar effectiveness of NSAIDs to colchicine or CS. The guidelines made different recommendations regarding dietary and lifestyle interventions. Similar to the ACR and EULAR guidelines [1, 3], the BSR guideline made detailed recommendations about specific foods that should be consumed or avoided. In contrast, the NICE guideline advised following a healthy, balanced diet based on there being insufficient evidence that any specific diet prevents flares or lowers urate. Both guidelines advocated a treat-to-target approach to ULT; however, the target serum urate level differed. The BSR guideline recommended an initial target serum urate level of <300 µmol/l (5 mg/dl) for all people receiving ULT. Once remission has been achieved, the ULT dose can be adjusted to maintain a target level of <360 µmol/l (6 mg/dl) to avoid theoretical neurodegenerative adverse effects associated with a very low serum urate level. In the NICE guideline, the target serum urate level was <360 µmol/l (6 mg/dl), with a lower target of <300 µmol/l (5 mg/dl) considered in those with tophi, chronic gouty arthritis or ongoing frequent flares, aligning the target level in the UK with the ACR and EULAR gout guidelines [1, 3]. The NICE guideline committee identified no high-quality evidence comparing different serum urate targets and felt that a target of <360 µmol/l (6 mg/dl) was more achievable and would incur lower costs, but recognized that a lower target was more appropriate for people with more severe gout. The guidelines also made differing recommendations about choice of ULT. The BSR guideline followed the 2008 NICE technology appraisal of febuxostat and recommended allopurinol as the first-line ULT [8], with febuxostat being a second-line option in those intolerant of allopurinol or in whom renal disease prevents sufficient dose escalation to achieve the target serum urate level. The NICE guideline committee undertook a costing analysis in view of the substantial reduction in the cost of febuxostat since the previous cost–utility analysis [9]. It also considered that febuxostat is more easily titrated than allopurinol because of having only two licensed doses and that adherence might be better because of once-daily dosing, although it also causes more flares than allopurinol, meaning that more people would need prophylaxis. It concluded that there was insufficient evidence to recommend allopurinol or febuxostat ahead of the other, reaching a recommendation that allopurinol or febuxostat can be used first line, replacing and updating its 2008 febuxostat technology appraisal [8]. Finally, the BSR guideline included recommendations about uricosuric drugs, which the NICE guideline did not consider because sulfinpyrazone, probenecid and benzbromarone are unlicensed in the UK and therefore cannot be included in a NICE guideline.

**Table 1.** Key differences between the 2017 British Society for Rheumatology and 2022 National Institute for health and Care Excellence gout guideline recommendations [6, 7]

Topic	BSR 2017 [6]	NICE 2022 [7]
NSAID, colchicine or CS for flares	NSAID or colchicine first line, dependent on patient preference, renal function and co-morbidities Oral/i.m. CS is an alternative if NSAIDs or colchicine are not tolerated	NSAIDs, colchicine or oral CS are first line, taking account of the person’s co-morbidities, co-prescriptions and preferences
Diet and lifestyle	Encourage a well-balanced diet Avoid sugar-sweetened soft drinks, fructose and excessive intake of alcoholic drinks and high-purine foods Skimmed milk, low-fat yoghurt, soy beans and vegetable sources of protein, and cherries should be encouraged	Insufficient evidence that any specific diet prevents flares or lowers serum urate levels. Advise following a healthy, balanced diet
Target serum urate level	≤300 µmol/l (5 mg/dl) Once symptom free, ULT dose can be adjusted to maintain the serum urate ≤360 µmol/l (6 mg/dl) to avoid possible adverse effects associated with a very low serum urate	<360 µmol/l (6 mg/dl). Consider <300 µmol/l (5 mg/dl) if: • tophi or chronic gouty arthritis • ongoing frequent flares, despite having a serum urate level <360 µmol/l (6 mg/dl)
Xanthine oxidase inhibitors	Allopurinol recommended as first-line treat-to-target ULT Febuxostat recommended as second line if allopurinol is not tolerated or renal impairment prevents allopurinol dose escalation	Allopurinol or febuxostat recommended as first-line treat-to-target ULT, taking into account the person’s co-morbidities and preferences
Uricosurics	Uricosuric agents can be used if xanthine oxidase inhibitors are not tolerated or are ineffective	Not considered

BSR: British Society for Rheumatology; CS: corticosteroids; NICE: National Institute for health and Care Excellence; ULT: urate-lowering therapy.

Differences between the BSR and NICE gout guideline recommendations arise from new evidence (flare management), insufficient high-quality direct evidence (diet/lifestyle, target serum urate level and choice of ULT), practicality and feasibility (target serum urate level and choice of ULT) and changing drug costs (choice of ULT). The NICE guideline is more up to date than the BSR guideline and should be considered the first-line guidance in the UK for managing most people with gout. However, the BSR guideline contains advice to guide management in special circumstances, such as chronic kidney disease, severe refractory tophaceous gout and pregnancy, in addition to making recommendations about drug dosing and use of uricosuric drugs, particularly when xanthine oxidase inhibitors are not tolerated or ineffective (Supplementary Table S1, available at *Rheumatology Advances in Practice* online). Although there are no plans currently to update the BSR guideline, it remains a helpful resource to guide management in more complex clinical situations. Both guidelines therefore have a place to guide clinicians to improve the management of this common but often poorly managed condition.

## Supplementary Material

rkae007_Supplementary_Data

## Data Availability

There are no new data associated with this article.
