# Supplementary material for: Diagnosis and management of gout: are the British Society for Rheumatology and National Institute for Health and Care Excellence guidelines both needed?
Source: Rheumatol Adv Pract. 2024 Jan 22;8(1):rkae007. doi: 10.1093/rap/rkae007 (PMC10809909; doi:10.1093/rap/rkae007)
Supplement: rkae007_Supplementary_Data [file rkae007_supplementary_data.docx]

**Supplementary Table S1:** Strengths of the 2017 BSR and 2022 NICE gout guideline recommendations [6,7]

| **BSR 2017 [6]** | **NICE 2022 [7]** |
| --- | --- |
| Robust transparent guideline development methods | Robust transparent guideline development methods |
| Multidisciplinary guideline development group including people with lived experience of gout | Multidisciplinary guideline development group including people with lived experience of gout |
| Included recommendations for drug dosing | Included recommendations for both diagnosis and management |
| Included recommendations for use of uricosuric drugs | Formally assessed cost-effectiveness of recommendations |
| Included specific advice for management of gout in:   - chronic kidney disease - severe, refractory tophaceous gout - pregnancy | More up-to-date (published 2022) |
| BSR, British Society for Rheumatology; NICE, National Institute for health and Care Excellence | |
